# Letter from J. H. Foster, M. D., Surgeon Dentist

**Published:** 1841-09

**Authors:** J. H. Foster


### ARTICLE V.

*The following letter from* J. H. FOSTER, M. D., Surgeon Dentist was then read by the Recording Secretary.

*New York, August* 7, 1841.

MR. PRESIDENT AND GENTLEMEN:

Having had the honour of being selected at the last annual meeting one of the members, to prepare a Thesis, to be read at the ensuing annual meeting of the Society, I deem it my duty to apologize to you, for neglecting it. When I found the time had almost imperceptibly nearly elapsed, I resolved to devote a part of the present week to this object, but my professional duties have called for my attention to an unusual degree, and I find I am compelled to throw myself upon your indulgence.

There is a question which I wish to propose for the consideration of the society, and I trust I shall not be considered egotistical, in presenting my own opinions in regard to it. If they chance to differ with those of many of my professional brethren, I hope at least I may have the credit of being considered sincere. My only object is to arrive at the truth, by exciting attention to the subject, and if I am wrong I am open to conviction.

The question is this: Are there cases, where it is essentially advisable and important that teeth should be filled, in which gold foil cannot be used, and other articles be substituted so as to preserve the teeth a sufficient time to compensate for the operation?

My own opinion is, that in all cases in which a tooth is worth the attempt to preserve it, it may be filled with gold, however difficult of access the cavity may be, and however tedious and laborious may be the operation of excavating and filling, and that when so filled, the chance is that the tooth will last much longer, than when any other article is used.

I have always believed, and have had no reason yet to doubt, that a tooth can be filled with gold if it can be filled at all. I know there are those who contend that in certain cases, owing to the extreme difficulty of obtaining access to the cavity, it is impossible (to use a common phrase) "to make a gold filling stay in," and that they can put in something else which will remain. And again, that the teeth are sometimes so extremely sensitive after having been excavated, that they will not bear the pressure consequent upon packing a gold filling, and that in such cases tin foil, or some other article, (the mineral succedaneum perhaps,) will answer a better purpose. I am free to declare that I do not place any faith in this; I do not believe there can be a cavity so situated that a gold filling cannot be placed in it, if any other can; and I am equally firm in the belief, that any tooth which will bear to have tin foil, or any other malleable metal packed into it sufficiently hard to remain, will not be too sensitive to support a solid gold filling, if the operation is properly performed, and the proper precautions taken which the peculiar circumstances of the case may require. If I can be shown cases affording full proof to the contrary, I may be induced to change my views upon the subject; but so long as cases are continually coming under my observation, in which I see teeth rapidly wasting away, which have been filled with tin, and which the patient was told at the time of the operation could not be filled with gold, and I feel satisfied they might at that time have been preserved by the use of gold, I cannot but feel that I am justified in my opinions. One of many such cases I will cite as an example. A gentleman and lady called on me about two weeks since for advice in regard to a molar tooth, saying they had no doubt I should advise extraction. On examination I found it to be the only upper molar tooth left; the opponents were sound, and I determined to preserve it if possible. There was a large cavity in the centre of the tooth, with a tin foil filling remaining in it, the wall on one side of which was partially broken away, and the other edges rough and irregular. I filed down the surface until I found the edges of the cavity sufficiently strong to support a filling. I then commenced excavating, and although the interior of the tooth was much softened, and the diseased portion highly inflamed, I removed it effectually, without reaching the nerve, and could cut in any part of the cavity upon the healthy bone without pain to the patient. I then packed into this cavity, (which I have no hesitation in saying, was at least one-third deeper, and the circumference nearly twice as great, as when the tin foil was originally placed,) as solid a gold filling as I should place in an ordinary cavity. I have seen the patient twice since, and although I sometimes feel doubtful in such cases in regard to the permanency of the operation, yet in this case I have no doubt the tooth will remain serviceable for many years. I have entered into these particulars on account of the fact, that a gentleman of considerable eminence in the profession, when he filled this same tooth three years before, had declared that it was impossible for the tooth to be filled with gold at that time. Such cases are by no means isolated; I have no doubt most of you have often met with them. But the question has been put to me, by gentlemen of the profession, why use so costly an article, and put the patient to such an expense, in these extreme cases, when there is an uncertainty whether the operation will prove serviceable? In an answer, that if a tooth can be preserved for one, two, or three years by filling it with tin, there is a chance, nay a strong probability, that a gold filling will preserve it much longer, and there are no cases, which redound more to the credit of a practitioner in our profession, than those which are considered doubtful, and which his operation proves successful in the preservation of. I have often found cavities partly filled with tin, and finished off with a shallow gold filling, and when it has become necessary to refill the teeth, and the patient is made aware of the artifice which has been practised, the operator reaps the reward of it.

I can only regret that there are members of the profession, who rank high in point of reputation in the community, who will thus incur the risk of destroying a good name. I do not wish to impugn any man's motives in such a course of practice, he can do no one a greater injury than he does himself: my only desire is that the truth may be known, and the attention of the society awakened to these malpractices, in regard to the most useful and important operation of dental surgery. I have never since I commenced practice, used a single particle of tin foil, or any other malleable metal or compound, for filling teeth; gold foil of the finest quality I consider to be the surest and best article in all cases, and when I find a tooth in which I cannot use it, it is time for the operation of extraction.

Teeth are often filled with some of these articles merely for the sake of preserving them a few months, because the patient is unwilling to submit to the operation of extraction, when it is manifestly the duty of the operator to urge the necessity and importance of it. Patients who have confidence in their practitioner, will readily submit to this operation if his judgment approves it, by his appealing to their reason and reflection.

There are too many timid practitioners, who shrink from this operation, and are ready to embrace any alternative rather than perform it, and they will sometimes even tell their patients that it is inadvisable or extremely dangerous, and send them off with some inefficient application. It is certainly much less difficult to persuade a patient to decline an operation than to submit to one, and he who through culpable timidity refuses to perform it, when he knows it to be his duty, is no more qualified for the practice of his profession, than he who is ignorant of the fundamental principles of it.

I trust, gentlemen, that you will excuse these remarks thus hastily penned; and I hope the day will yet come when malpractices shall be corrected, the darkness of ignorance dispelled, and commensurate with the talents and worth of its professors, its extensive bearing upon health, and its happy influence upon society, dental surgery shall assume a corresponding rank among the medical sciences. Respectfully, your obd't serv't,

J. H. FOSTER.

---

In publishing other parts of the proceedings of the Society, we shall extract from the minutes, such portions only as we deem of general interest to the profession.—*Eds.*

